# Effect of Transcutaneous Application of Carbon Dioxide on Wound Healing, Wound Recurrence Rate and Diabetic Polyneuropathy in Patients with Neuropathic, Ischemic and Neuroischemic Diabetes-Related Foot Ulcers

**DOI:** 10.3390/life15040618

**Published:** 2025-04-07

**Authors:** Tomislav Bulum, Tamara Poljičanin, Anica Badanjak, Jelena Držič, Željko Metelko

**Affiliations:** 1 Clinical Hospital Merkur, University Clinic for Diabetes, Endocrinology, and Metabolic Diseases Vuk Vrhovac, Dugi dol 4a, 10000 Zagreb, Croatia; 2 School of Medicine, University of Zagreb, Šalata 3, 10000 Zagreb, Croatia; 3 Zagreb County Health Center, Ljudevita Gaja 37, 10430 Samobor, Croatia; 4 Polyclinic for Physical Medicine and Rehabilitation with Physical Therapy, Vascular Surgery, Neurology, Endocrinology, and Diabetology, Kalinovica 3, 10000 Zagreb, Croatia

**Keywords:** diabetic foot, diabetes-related foot ulcer, CO_2_ therapy, transcutaneous CO_2_ application, wound healing, distal symmetrical polyneuropathy, loss of protective sensation

## Abstract

(1) Background: Diabetes-related foot ulcers (DFUs) are a severe complication of diabetes mellitus (DM), with a five-year mortality rate of around 40%. Our study aimed to explore the effects of transcutaneous application of carbon dioxide (CO_2_ therapy) on DFU healing and recurrence rate, as well as diabetic polyneuropathy. (2) Methods: Adults with at least one chronic DFU were invited to undergo 20 50-min-long CO_2_ therapies within 4 weeks. After the completion of the last CO_2_ therapy, the effect of the therapies on wound healing and diabetic polyneuropathy was assessed, and 1 year later, we evaluated the incidence rate of DFU recurrence. (3) Results: Thirty-five subjects with DM and forty DFUs (ischemic: 15, neuropathic: 8, neuroischemic: 17) participated in our trial. After 4 weeks, 67.5% of all DFUs healed, whereas the reduction of the surface area of the unhealed wounds (74.0% ± 22.3%) was statistically significant. The restoration of protective sensations was also statistically significant. All unhealed wounds received standard care and healed within 2 additional weeks. The recurrence rate after 1 year was 17.5%. None of the patients required antibiotic treatment, hospitalization, or amputation. (4) Conclusion: CO_2_ therapy is a promising therapeutic intervention for treating DFUs and improving diabetic polyneuropathy.

## 1. Introduction

Diabetes-related foot ulcers (DFUs) represent one of the most serious complications of diabetes mellitus (DM). It is reported that out of the estimated 537 million people with DM in 2022 (projected to reach 643 million people by 2030), 19 to 34% will develop a DFU [1]. Given the fact that there is an alarmingly high percentage (45%) of people with undiagnosed DM [2], the burden associated with DFUs, such as quality of life, mortality, and financial burden, is even more significant.

The International Working Group on the Diabetic Foot (IWGDF) Guidelines on the prevention and management of diabetic-related foot disease [3] represent the state-of-the-art document for the treatment of DFUs. These guidelines provide 29 separate recommendations and several conditional supportive recommendations. However, due to the rising incidence, costs of DFU management, and high amputation rates, there is a big necessity for identifying wound healing-enhancing interventions for DFUs [4]. An example of such an intervention is carbon dioxide (CO_2_) therapy. Generally speaking, such therapy denotes (1) a transcutaneous application of gaseous CO_2_ or CO_2_ dissolved in water (the latter also known as a CO_2_ or CO_2_-enriched bath) or (2) subcutaneous injection of gaseous CO_2_ for a specific therapeutic purpose. Among these several variations of CO_2_ therapy, the transcutaneous (also termed transdermal or percutaneous) application of CO_2_ seems to be the most promising due to its non-invasiveness and ease of use. In addition, it poses no inflammation risk. Transcutaneously applied CO_2_ acts as a vasodilator in cutaneous microcirculation (increases tissue perfusion) [5], while at the same time causing the “Artificial Bohr effect,” i.e., CO_2_ that transcutaneously enters one’s blood circulation causes oxygen dissociation from oxygenated hemoglobin [6]. The positive effect of CO_2_ therapies on cutaneous microcirculation has also been demonstrated by neoangiogenesis via vascular endothelial growth factor (VEGF) gene expression [7,8], increased Lasser-Doppler flux in healthy subjects during a single CO_2_ therapy [9], and improved endothelial and neurogenic regulation of cutaneous blood flow in subjects with DFUs after prolonged treatment with CO_2_ therapies [10]. There are numerous reported indications of CO_2_ therapies [5], with the most promising results in wound treatment [9,11,12,13,14,15]. Providing that microcirculatory function plays a crucial role when it comes to the healing of DFUs (although this role has not yet been fully understood [16,17,18], the presented mechanisms of action of transcutaneously applied CO_2_ support the use of CO_2_ therapies for the treatment of DFUs. Finally, the transcutaneous application of CO_2_ also supports muscular strength recovery [7], which is relevant for the treatment of DFUs, since protective strategies are most often preferred over exercise [19] in patients with DFUs.

The most common transcutaneous CO_2_ application method is the application of gaseous CO_2_. In this method, the lower part of the body (i.e., below the waist) of patients is exposed to medical-grade gaseous CO_2_ for a certain amount of time. Recently, an alternative to the transcutaneous application of gaseous CO_2_ has also been introduced—the transcutaneous application of “CO_2_ paste” [13]. The advantage of this application method relates to its possible use on the head and neck area [20]. Although this method is not beneficial in the treatment of DFUs, it clearly shows that the field of CO_2_ therapies is evolving due to its undeniable potential, particularly in promoting wound healing.

To further study and understand the effect of CO_2_ therapies in the treatment of DFUs, we set the goal of assessing the effect of repeated CO_2_ therapies on the healing of DFUs and their recurrence, together with their effect on diabetic distal sensorimotor polyneuropathy (DSPN). Our clinical trial was designed in a way to raise the certainty of evidence in order for CO_2_ therapy to be widely used, especially due to its significant role in improving microvascular function [10], which is its main advantage compared to other interventions with mainly local effects on DFUs. Our study aimed to explore the effects of transcutaneous application of gaseous CO_2_ therapy on wound healing, wound recurrence rate, and DSPN in patients with DFUs.

From this point on, the term CO_2_ therapy is used to refer exclusively to the transcutaneous application of gaseous CO_2_.

## 2. Materials and Methods

### 2.1. Clinical Trial Protocol and Objectives

The objective of our clinical trial was to assess the effect of twenty (20) CO_2_ therapies on the healing of DFUs with neuropathic, ischemic, and neuro-ischemic etiologies. Additionally, the trial also aimed to assess the effect of improving protective sensation in the participating subjects. The trial took place at the Polyclinic for Physical Medicine and Rehabilitation with Physical Therapy, Vascular Surgery, Neurology, Endocrinology, and Diabetology (Zagreb, Croatia) and lasted for 8 months, starting in Q4 2022 and ending in Q3 2023. The inclusion criteria were as follows: subject of any gender, aged from 18 to 85 years, with DM (type 1 or 2) and at least one chronic DFU regardless of its etiology, and willingness to undergo the complete set of CO_2_ therapies (subjects were not hospitalized but underwent outpatient treatment). The exclusion criteria were the contraindications of CO_2_ therapies: severe cardiac, renal, or pulmonary disease, deep vein thrombosis, malignant diseases, and progressive infections or systemic infection with increased inflammatory markers [21]. All subjects interested in participating in the study and meeting all inclusion criteria were given a complete overview of the clinical trial and were able to ask any questions. Written informed consent was obtained from the subjects after adequate information about the clinical trial was provided to them, allowing each subject to make an informed decision regarding their participation in the clinical trial. Before the first CO_2_ therapy, anamnesis was taken, selected diagnostic tests were carried out, and each DFU was assessed by a physician. After the completion of the last CO_2_ therapy, selected diagnostic tests were repeated, and wounds were reassessed. After the completion of the trial, all subjects with unhealed wounds underwent standard-of-care (SOC) treatment. Finally, 1 year after the completion of the clinical trial, all participating subjects were involved in follow-up consultations with a physician to define the incidence rate for DFU recurrence.

### 2.2. Diagnostic Tests

We carried out the following procedures before the first CO_2_ therapy: (1) hemoglobin A1c (HbA1c) blood test, (2) ankle-brachial index (ABI) measurement on both legs using MESI ABPI MD (Mesi d.o.o., Ljubljana, Slovenia), (3) measurement of temperature on the plantar surface of the first toe on the foot with ulcer(s) using digital infrared thermometer Voltcfraft IR 800-20D (Voltcraft, London, UK), (4) 10 g Semmes Weinstein monofilament (SWM) examination, and (5) vibration sensation test.

To evaluate the loss of protective sensation, the SWM examination was conducted by testing the eight standard points on each foot (a total score of 16 denotes that no sensation was felt at any of the testing points). In the vibration test applied, a 128 Hz tuning fork on five standard points on each foot was used (a total score of 10 denotes that no sensation was felt at any of the testing points). The purpose of the SWM examination and vibration test was to assess the effect of CO_2_ therapies on DSPN. Before the presented procedures were carried out, participating subjects were instructed to lie barefoot on the examination table for 10 min to acclimate.

### 2.3. Wound Assessment

Before the first CO_2_ therapy, each DFU was (1) evaluated according to the Site, Ischemia, Neuropathy, Bacterial Infection, and Depth (SINBAD) classification [22], (2) given the Falanga wound bed score [23], and (3) measured (in cm^2^) using the non-contact 3D scanner DAVID SLS-2 (David Vision Systems GmbH, Koblenz, Germany). After the evaluation procedure was presented, each DFU was sharply debrided. After the completion of the last CO_2_ therapy, each DFU was first classified as healed or unhealed by a physician. For a DFU to be classified as healed, the following requirement had to be met: “The intact skin at a previous foot ulcer site, meaning complete epithelialisation without any drainage” [24]. The term epithelialisation refers to a process in which epithelial cells grow over a wound to cover the previously exposed surface. Each unhealed DFU was re-assessed by being given Falanga and SINBAD scores, and its surface was re-measured.

During the duration of the trial, the standard protocol was followed for wound care in all subjects and included the use of atraumatic wound dressings (changed every 2 days) and offloading shoes to offload the pressure on wounds.

### 2.4. CO_2_ Therapy

**Figure 1:**
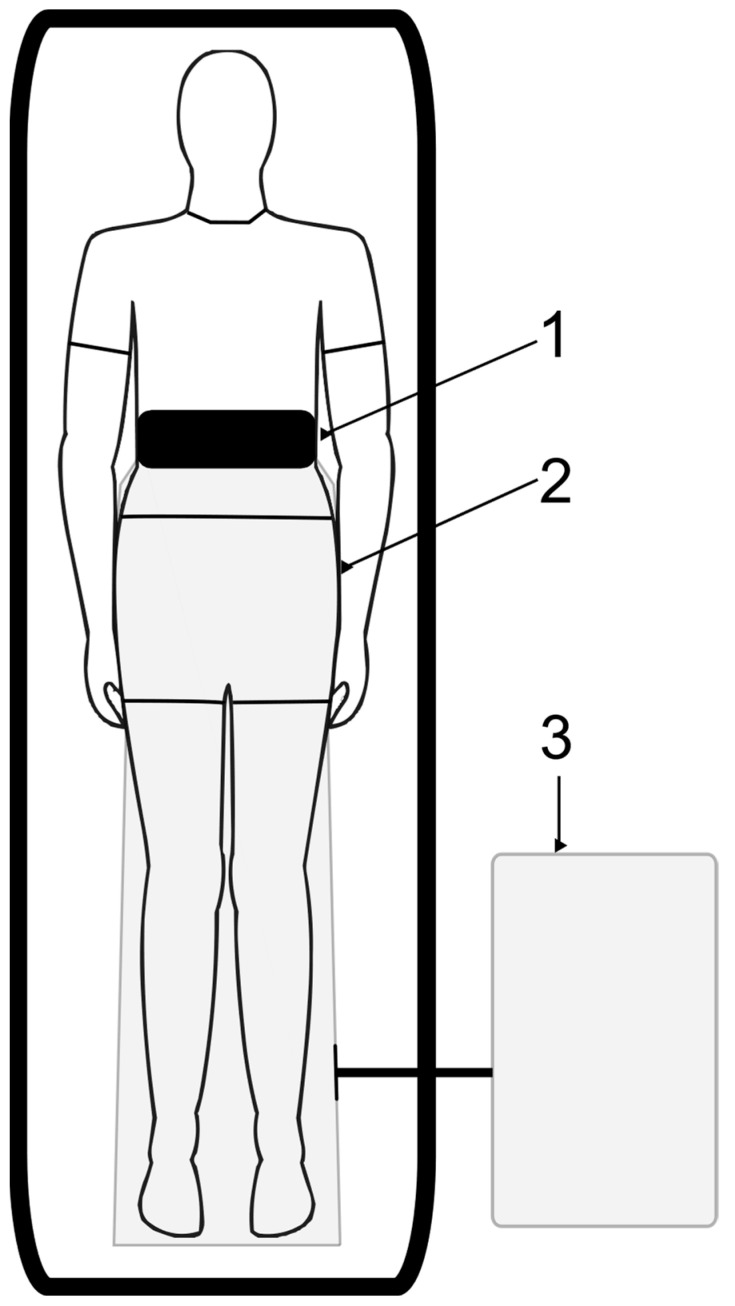
Schematic representation of a subject (in supine position) during CO_2_ therapy. 1—snug safety belt, 2—single-use biocompatible polyethylene bag, 3—PVR system.

CO_2_ therapies were carried out using the medical device PVR system (Derma Art d.o.o. and PVR med d.o.o., Brežice, Slovenia). The procedure was as follows: The lower part of the body of a participating subject was isolated into a single-use, biocompatible polyethylene therapeutic wrap, sealed at the waist. Then, the therapy wrap was filled with medical-grade CO_2_ using a dedicated medical device. After reaching a 99.9% concentration of CO_2_, CO_2_ therapy started and lasted for 50 min. During the entire duration of CO_2_ therapy, the subjects were in a supine position. After the therapy was completed, CO_2_ was pumped out of the wrap, and the wrap was removed (Figure 1). During and after each therapy, subjects were asked to describe potential side effects. In addition, the personnel carrying out the therapies paid attention to identifying potential adverse effects. Each subject underwent CO_2_ therapy once per working day for 4 consecutive weeks, reaching a total of 20 CO_2_ therapies by the end of the trial.

### 2.5. Terminology

The vocabulary used within our clinical trial and this article is aligned with foot-disease-related definitions set by the IWGDF [24], allowing the results of our clinical trial to be more easily comparable with other trials/research studies.

### 2.6. Statistical Analysis

Statistical analysis of the results was carried out using IBM SPSS Statistics 21.0 (IBM, New York, NY, USA). Numerical data are presented as (1) mean ± standard deviation or (2) median (minimum value–maximum value), while categorical variables are presented as frequencies. Normality of distribution was tested using the Shapiro-Wilks test, homogeneity of variance using the Levene test, differences between groups of independent continuous variables using the Mann-Whitney U test, differences between groups of dependent continuous variables using the Wilcoxon signed-rank test, occurrence of categorical conditions using chi-square and Fisher’s exact test (where indicated), and differences between baseline and after treatment continuous characteristics were compared using the Wilcoxon matched-paired test. The significance level (the probability of a type I error) was set to α = 0.05.

## 3. Results

A total of 35 subjects were enrolled in the clinical trial. None of the subjects who initially decided to enroll in the trial withdrew from it later. The characteristics of the participating subjects are shown in Table 1.

Table 2 provides the characteristics of DFUs of the participating subjects before the beginning of treatment with CO_2_ therapies. Two subjects had three DFUs, and one subject had two DFUs; therefore, the total number of wounds is larger than the number of participating subjects.

After the completion of 20 CO_2_ therapies in all participating subjects, a total of 27 wounds in 24 subjects were healed, which translates to 67.5% of all wounds. None of the subjects reported any side effects during or after any of the CO_2_ therapies; the personnel responsible for carrying out CO_2_ therapies did not identify any such effect in any subject. Next, there was no indication of antibiotic treatment, amputation, or hospitalization in any of the patients during the clinical trial.

The characteristics of subjects with healed wounds and those with unhealed wounds, together with their results of diagnostic tests before and after the CO_2_ therapies, are shown in Table 3.

Table 3 shows that there is only one statistically significant difference between the two groups, i.e., the age (*p* = 0.036). In both groups, there was a statistically significant difference between the temperature on the plantar surface of the first toe on the foot with ulcer(s) before the first CO_2_ therapy (T_1_) and the temperature on the plantar surface of the first toe on the foot with ulcer(s) after the last CO_2_ therapy (T_2_; *p* < 0.001 for the group of subjects with healed wounds and *p* = 0.002 for the group of subjects with unhealed wounds).

In both groups, there was a statistically significant difference between the wound surface before the start of the first CO_2_ therapy (P_1_) and the wound surface after the completion of the last CO_2_ therapy (P_2_; *p* < 0.001 for the group of subjects with healed wounds and *p* = 0.001 for the group of subjects with unhealed wounds). The wound assessment results for both groups before and after CO_2_ therapies are shown in Table 4.

The results of the follow-up consultations with a physician after 12 months are shown in Table 5. A total of 7 wounds recurred, resulting in a recurrence rate of 17.5%, with 71.4% of wounds recurring on the same foot and 28.6% on the contralateral foot. Nevertheless, there were no complications in any of the subjects that required antibiotic treatment, hospitalization, or amputation.

## 4. Discussion

The achieved wound healing rate—27 out of 40 wounds (67.5%) healed in 4 weeks—strongly supports the usage of CO_2_ therapies as a crucial microvascular-supporting intervention in the treatment of DFUs. This result is even more prominent given the fact that the success rate was independent of wound characteristics, glycaemic control, and the presence of peripheral arterial disease (PAD). In addition, during and after the completion of the clinical trial, none of the subjects underwent any additional CO_2_ therapies; those with unhealed wounds just underwent standard wound care, with their DFUs being examined and wound dressings replaced twice a week. From the perspective of wound etiology, 37.5% of wounds were neuropathic, 20% ischemic, and 50% of aneuroischemic origin. This distribution follows the distribution reported in the literature [25], which means that the sample studied sufficiently represented the population. The largest number of wounds healed was of ischemic origin (44.4%), followed by those of neuroischemic origin (37%), whereas the lowest number of healed wounds was those of neuropathic origin (18.5%). The distribution of unhealed wounds was as follows: ischemic 23.1%, neuropathic 23.1%, neuroischemic 53.8%. However, there were no statistically significant differences (*p* = 0.418) in wound etiology between the two groups. The potential effect of glycaemic control on wound healing was assessed by the HbA1c test; even though the HbA1c was slightly larger in subjects with unhealed wounds, the difference was not statistically significant. Lastly, the measurement of ABI allowed us to identify the potential presence of PAD. Based on the difference (although not statistically significant) between the subjects with healed wounds and those with unhealed wounds, it suggests that the healing process in patients with PAD is slower than in patients without this condition. However, PAD still has some effect on the healing process. Out of 11 subjects with unhealed wounds, PAD was present in 5 subjects (45.5%), whereas in the group of subjects with healed wounds, PAD was present in 6 out of 24 subjects (25%). Nevertheless, the wound healing process of wounds in patients with PAD was still evident—the median value before the first CO_2_ therapy was 1.8 cm^2^ (0.7–3.2 cm^2^), whereas after the last CO_2_ therapy, the median value was 0.3 cm^2^ (0.1–1 cm^2^). Please note that given such a small sample size, the computation of statistical significance would not be appropriate, as it would increase the risk of the result being unreliable and misleading. This indicates that CO_2_ therapies are efficient in patients with PAD. The effect of CO_2_ therapies on PAD itself using the ABI measurement method, unfortunately, cannot be evaluated, since this method does not assess the collateral blood flow, which is affected positively by CO_2_ [26]. Furthermore, the measurement of the ABI index in PAD patients does not predict limb outcome; it is the microcirculatory function that is crucial in decreasing amputation rates in patients with PAD [27]. This makes CO_2_ therapy and its efficacy on microcirculation even more advantageous than adjunct therapies that do not affect microcirculation, especially when considering that PAD is up to seven-fold more prevalent in people with DM compared to those without it (with rates ranging from 9% to 55% based on several hospital studies) [28].

The only relevant statistically significant difference between the subjects with healed wounds and those with unhealed wounds before the first CO_2_ therapy was the wound surface. Although the mean wound surface was larger in the group with subjects with unhealed wounds, the effect on their wound healing is evident (see the next paragraph for more details). The other statistically significant difference between the two groups before the first CO_2_ therapy was age, with the mean age of subjects with healed wounds being larger than that of the subjects with unhealed wounds. Given the fact that the process of wound healing is altered in aged individuals in a way that leads to impaired wound healing [29] the effect of CO_2_ therapies is even more promising.

In none of the patients with unhealed wounds did the Falanga wound bed score and SINBAD classification score deteriorate after the completion of all CO_2_ therapies compared to the scores before the first CO_2_ therapy. Furthermore, the surfaces of all unhealed wounds were reduced after the completion of the last CO_2_ therapy—the healed surface was 2.17 ± 2.27 cm^2^ (average ± standard deviation), which translates to 74.0% ± 22.3% of the initial wound surface being healed. The median wound healing rate, expressed as an absolute surface healed per day in subjects with unhealed wounds, was 0.07 cm^2^/day (with a minimum rate of 0.01 cm^2^/day and a maximum rate of 0.34 cm^2^/day), or, expressed as the percentage of initial surface healed per day, 3.2%/day (with a minimum rate of 1.2%/day and a maximum rate of 3.7%/day). Although these two rates are limited when it comes to comparing healing rates of wounds with different initial sizes [30], they are still indicative of the effect of CO_2_ therapies.

The recurrence rate of 17.5% within the first year (with a zero-recurrence rate within 6 months) is a very promising outcome, considering that the recurrence rate of DFUs within the first year after wound healing is 40% [1]. The investigation into the potential causes of wound recurrence showed the following information: (a) two subjects did not follow the recommendations related to therapeutic footwear and were daily exposed to extreme levels of stress due to their job conditions; (b) two subjects underwent a below-knee amputation before the clinical trial and had; (c) one patient had a high body mass index (BMI) of (36.9 kg/m^2^) and Charcot arthropathy; (d) two patients had thickened nails due to onychomycosis accompanied by hammer toe deformity. The potential factors behind the wound recurrence in the subjects presented are (1) biomechanical factors (increased plantar stress associated with barefoot and in-shoe mechanical stress), (2) vascular factors (presence of PAD), and (3) foot deformity factors. All these factors are well aligned with the ones reported in the literature [1].

The effect of CO_2_ therapies on DSPN is also evident—there are statistically significant differences in SWM test scores in both groups before and after the treatment with CO_2_ therapies (*p* < 0.001). In the case of the vibration test score, the difference is statistically significant for the group of subjects with healed wounds (*p* = 0.021), whereas this is not the case for the group of subjects with unhealed wounds (*p* = 0.29). Given the fact that the presumed underlying pathophysiology of DSPN is metabolic-microvascular-hypoxic, with the microvascular component associated with microvascular changes in peripheral nerves [31] and the severity of DSPN associated with the severity of microvascular changes [31], the effect of CO_2_ therapies on DSPN is explainable. Improvement of DSPN after treatment with CO_2_ therapies has also been reported by other studies [21].

From the perspective of the core outcome set (COS) for studies assessing interventions for DFUs, recently developed by Staniszewska et al. [32], our study addressed all outcomes (wound healing, time to healing, new/recurrent ulceration, infection, major amputation, minor amputation, and mortality) except for health-related quality of life, the measurement of which has also been identified as challenging [32]. We hope that the following COS will facilitate the assessment of the comparative effectiveness of CO_2_ therapy with other interventions.

In comparison with the current SOC consisting of pressure relief, debridement, infection management, and revascularization (when indicated), CO_2_ therapies seem to be rather superior. SOC is usually provided for 4 weeks since studies have shown that DFUs not reducing in size by 50% within this period are less likely to heal by 12 weeks [33]. In our study, we have shown that the period of 4 weeks was long enough for most of the wounds to heal, whereas the minimum reduction in size for any of the unhealed wounds was 43%. Nevertheless, even this particular wound healed within 6 weeks after the last CO_2_ therapy and did not recur after 1 year.

Our clinical trial and its results have some potential limitations. First, the small number of patients and DFUs included in the study is the major limitation, which prevents the generalization of these results to populations with DM. Second, the number of wounds was larger than the number of participating subjects since some of the subjects had more than one wound present. Third, all patients were Caucasian and of European origin, so the findings may not be generalizable to populations of different ethnicities. Despite the limitations presented, the findings of this study contribute significantly to the field of CO_2_ therapies for DFU treatment and the improvement of protective sensation.

Based on the promising results of our clinical trial and other studies involving CO_2_ therapies, it should be of great importance for physicians and researchers working with CO_2_ therapies to form a working group that would define the common terminology and protocols for CO_2_ therapies to facilitate the comparison between different studies as well as help achieve recognition of CO_2_ therapies among the IWGDF group. Our future work is related to conducting a post-healing follow-up study within 1 year, 2 years, and 5 years after the end of the clinical trial by carrying out annual consultations with participating subjects. With this follow-up study, we want to assess the prolonged effect of CO_2_ therapies on wound recurrence and complications associated with DFUs treated with CO_2_ therapies. In the longer term, we aim to carry out a clinical trial that would overcome the limitations of this trial, i.e., include a larger number of patients of different ethnicities, allowing for the generalization of the effect of CO_2_ therapies on DFUs and DSPN.

## 5. Conclusions

DFUs are a severe complication of DM, with a high five-year mortality rate of around 40%. Between 19% and 34% of patients with DM will develop a DFU, impacting their quality of life, increasing mortality risk, and adding to the financial burden. Our single-arm clinical trial suggests that repeated transcutaneous application of gaseous CO_2_ shortens wound healing time and leads to wound closure and improved protective sensation (DSPN) in patients with DFUs without causing adverse effects. Our findings support the use of CO_2_ therapy for diabetic foot treatment, particularly due to its non-invasive, painless mechanism of action, its ability to avoid patient stress, and its pharmacologically neutral profile. Given the extensive pharmacological treatments often required for these patients, CO_2_ therapy emerges as a valuable option that enhances microcirculatory function, promotes wound healing, and supports sensory nerve regeneration without adding to the systemic medication burden.

## Figures and Tables

**Table 1:** The characteristics of the subjects participating in our clinical trial together with the results of the diagnostic tests.

Characteristic	Value
No. of subjects	35: M (28), F (7)
BMI [kg/m^2^]	29.4 (22.6–44.2)
DM	Type 2 (35)
HbA1c [%]	7.3 (5.8–11.4)
ABI [/] *	L: 0.97 ± 0.23 ^a^ R: 0.96 ± 0.23 ^b^
T [°C] *	27.2 ± 3.3
SWM score	16 (0–16)
Vibration test score	4 (0–10)

Legend: M–male, F–female, BMI–body mass index, HbA1c–Hemoglobin A1c, DM–diabetes mellitus, ABI–Ankle-Brachial Index, L–left leg, R–right leg, T–temperature on the plantar surface of the first toe on the foot with ulcer(s), SWM—Semmes Weinstein monofilament test. ^a^ The result for 26 subjects, ABI was unmeasurable in 9 patients; ^b^ The result for 27 subjects, ABI was unmeasurable in 8 patients. Values are expressed as median (minimum–maximum value) or number of patients unless stated otherwise. * Values expressed as a mean ± standard deviation.

**Table 2:** Characteristics of the ulcers of the participating subjects before the onset of the first CO_2_ therapy.

Characteristic	Value
No. of wounds	40
Etiology	I (15), N (8), NI (17)
Ulcer age [month]	2 (1–12)
Falanga score	B (24), C (16)
SINBAD score	1 (1), 2 (6), 3 (7), 4 (10), 5 (13), 6 (3)
P [cm^2^]	1.5 (0.3–11.1)
Amputation history	Yes (10) ^a^, No (25)

Legend: I—ischemic, N—neuropathic, NI—neuroischemic, B—50–100% granulation tissue, C—<50% granulation tissue, P—wound surface, SINBAD—Site, Ischemia, Neuropathy, Bacterial Infection, and Depth classification. ^a^ Two patients had a history of below-knee amputations. Values are expressed as median (minimum–maximum value) or number of patients.

**Table 3:** Characteristics of subjects with healed wounds (Group 1) and subjects with unhealed wounds (Group 2) together with the diagnostic test results before the first CO_2_ therapy and after the completion of the last CO_2_ therapy.

	Group 1	Group 2	*p*
Group size	24: M (19), F (5)	11: M (9), F (2)	0.856
Age [year]	74 (49–83)	67 (41–79)	0.036
BMI [kg/m^2^]	29.2 (22.6–41.7)	29.4 (22.6–44.2)	0.636
DM type	Type 2 (24)	Type 2 (11)	/
HbA1c [%]	7.3 (5.8–11.4)	7.3 (6.2–10.8)	0.540
ABI [/] *	L: 0.93 ± 0.26 ^a^ R: 0.97 ± 0.25 ^b^	L: 1.02 ± 0.10 ^c^ R: 0.96 ± 0.16 ^d^	0.285 0.589
T_1_ [°C] *	27.6 ± 3.6	26.5 ± 2.6	0.311
T_2_ [°C] *	31.3 ± 1.6	30.6 ± 2.0	0.398
SWM score 1	16 (0–16)	16 (3–16)	0.793
SWM score 2	8 (0–16)	10 (0–14)	0.370
Vibration test score 1	4 (0–10)	4 (0–10)	0.224
Vibration test score 2	2 (0–10)	2 (0–8)	0.958

Legend: Group 1—Group of subjects with healed wounds, Group 2—Group of subjects with unhealed wounds, *p*—*p*-score, M—male, F—female, BMI—body mass index, DM—diabetes mellitus, HbA1c—Hemoglobin A1c, ABI—Ankle-Brachial Index, L—left leg, R—right leg, T_1_—temperature on the plantar surface of the first toe on the foot with ulcer(s) before the first CO_2_ therapy, T_2_—temperature on the plantar surface of the first toe on the foot with ulcer(s) after the last CO_2_ therapy, SWM score 1—score of the Semmes Weinstein monofilament test carried out before the first CO_2_ therapy, SWM score 2—score of the Semmes Weinstein monofilament test carried out after the last CO_2_ therapy. ^a^ The result for 18 subjects, ABI was unmeasurable in 6 patients; ^b^ The result for 21 subjects, ABI was unmeasurable in 3 patients; ^c^ The result for 9 subjects, ABI was unmeasurable in 3 patients; ^d^ The result for 6 subjects, ABI was unmeasurable in 5 patients. * Value expressed as a mean ± standard deviation. Values are expressed as median (minimum–maximum value) or number of patients unless stated otherwise.

**Table 4:** Results of wound assessment after the completion of all CO_2_ therapies.

	Group 1	Group 2	*p*
Etiology	I (12), N (5), NI (10)	I (3), N (3), NI (7)	0.418
Ulcer age [month]	2 (1–12)	2 (1–12)	0.887
Falanga score 1	B (18), C (9)	B (6), C (7)	0.215
Falanga score 2	wounds healed (27)	A (12), B (1)	/
SINBAD score 1	1–4 (17); 5–6 (10) 1 (1), 2 (5), 3 (6), 4 (5), 5 (8), 6 (2)	1–4 (7); 5–6 (6) 1 (0), 2 (1), 3 (1), 4 (5), 5 (5), 6 (1)	0.581
SINBAD score 2	wounds healed (27)	1–4 (13), 5–6 (0) 1 (3), 2 (2), 3 (7), 4 (1), 5 (0), 6 (0)	/
P_1_ [cm^2^]	2.1 (0.3–11.1)	1.2 (0.3–4.1)	0.031
P_2_ [cm^2^]	0 (0–0)	0.2 (0.1–2.9)	<0.001
Amputation history	Yes (5) ^a^	Yes (3)	0.774

Legend: Group 1—Group of subjects with healed wounds, Group 2—Group of subjects with unhealed wounds, *p*—*p*-score (comparison between groups), I—ischemic, N—neuropathic, NI—neuroischemic, *p*—*p*-score, B—50–100% granulation tissue, C—<50% granulation tissue, A—100% granulation tissue, SINBAD score 1—Site, Ischemia, Neuropathy, Bacterial Infection, and Depth classification score before the first CO_2_ therapy, SINBAD score 2—Site, Ischemia, Neuropathy, Bacterial Infection, and Depth classification score after the last CO_2_ therapy, P_1_—wound surface before the start of the first CO_2_ therapy, P_2_—wound surface after the completion of last CO_2_ therapy. ^a^ Two patients had a history of below-knee amputation. Values are expressed as median (minimum–maximum value) or number of patients.

**Table 5:** Results of the wound recurrence 12 months after the completion of the clinical trial.

	**No. of Recurred Wounds on the Same Foot**	**No. of Recurred Wounds on a Contralateral Foot**
Group 1	4	0
Group 2	1	2

Legend: Group 1—Group of subjects with healed wounds, Group 2—Group of subjects with unhealed wounds.

## Data Availability

The original contributions presented in this study are included in the article. Further inquiries can be directed at the corresponding author.

## Abbreviations

The following abbreviations are used in this manuscript:

ABI	ankle-brachial index
BMI	body-mass index
CO_2_	carbon dioxide
COS	core outcome set
DFU	diabetes-related foot ulcer
DM	diabetes mellitus
DSPN	diabetic distal sensorimotor polyneuropathy
HbA1c	hemoglobin A1c
IWGDF	The International Working Group on the Diabetic Foot
PAD	peripheral arterial disease
SINBAD	Site, Ischemia, Neuropathy, Bacterial Infection, and Depth
SOC	standard of care
SWM	Semmes Weinstein monofilament
VEGF	vascular endothelial growth factor
